# Publisher Correction: Disordered Supersolids in the Extended Bose-Hubbard Model

**DOI:** 10.1038/s41598-018-23002-4

**Published:** 2018-03-14

**Authors:** Fei Lin, T. A. Maier, V. W. Scarola

**Affiliations:** 1 Department of Physics, Virginia Tech, Blacksburg, Virginia 24061 USA; 2 Computational Science and Engineering Division and Center for Nanophase Materials Sciences, Oak Ridge National Laboratory, Oak Ridge, Tennessee 37831 USA

Correction to: *Scientific Reports* 10.1038/s41598-017-13040-9, published online 06 October 2017

This Article contains an error in Figure 4, where the key was omitted. The correct Figure 4 appears below as Figure 1.

**Figure 1:**
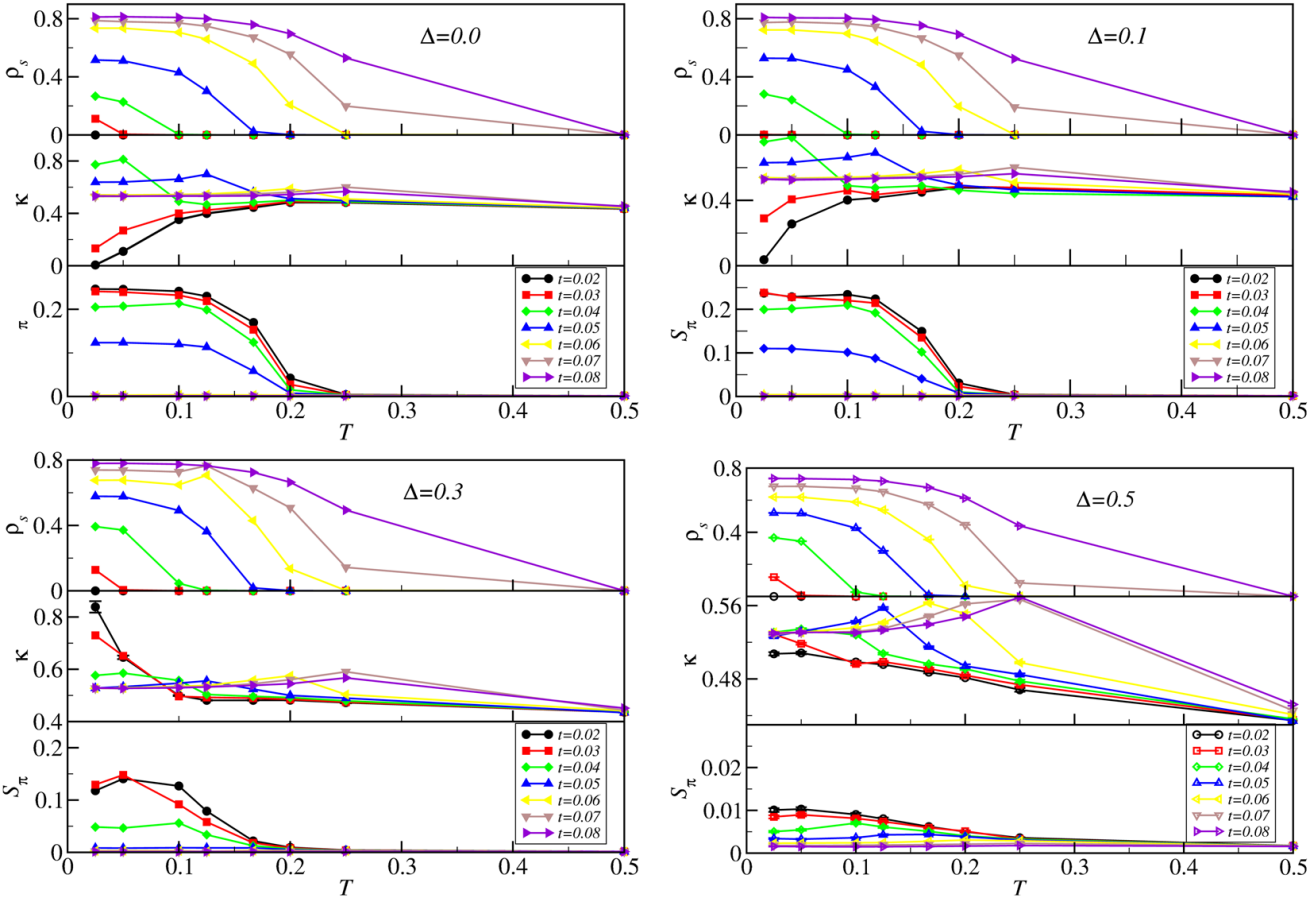
Order parameters versus temperature. Order parameters *ρ*_*s*_, *κ*, and *S*_*π*_ computed as functions of temperature *T* and hopping *t* for various disorder strengths using quantum Monte Carlo on Eq. (1) for *L* = 10. Here and in the following the error bars are smaller than the symbol size unless depicted otherwise and the lines are guides to the eye. We set *μ* = 0.7 and *V* = 1/*z* here. Parameters *t* and Δ are shown in the figure.

