# Supplementary material for: Disordered Supersolids in the Extended Bose-Hubbard Model
Source: Sci Rep. 2017 Oct 6;7:12752. doi: 10.1038/s41598-017-13040-9 (PMC5630629; doi:10.1038/s41598-017-13040-9)
Supplement: Supplementary file 1 — Supplementary Info [file 41598_2017_13040_MOESM1_ESM.pdf]

Supplemental Materials: "Disordered Supersolids in the Extended Bose-Hubbard Model"

[1]Fei Lin, [2]T. A. Maier, and [1,*]V.W. Scarola
[1]*Department of Physics, Virginia Tech, Blacksburg, Virginia 24061, USA*
[2]*Computational Science and Engineering Division and Center for Nanophase Materials Sciences, Oak Ridge National Laboratory, Oak Ridge, Tennessee 37831, USA and*
[*]*scarola@vt.edu*

Supplemental Materials: "Disordered Supersolids in the Extended Bose-Hubbard Model"

Mapping to the Attractive Fermi-Hubbard Model.

The attractive Fermi-Hubbard model is given by:

$$H_{\rm F} = -t^{\rm F}\sum_{\langle i,j\rangle,\sigma}\left(c^{\dagger}_{i,\sigma}c_{j,\sigma}+{\rm H.c.}\right) - |U^{\rm F}|\sum_i n^{\rm F}_{i,\uparrow}n^{\rm F}_{i,\downarrow} + V^{\rm F}\sum_{\langle i,j\rangle} n^{\rm F}_i n^{\rm F}_j - \sum_i \mu^{\rm F}_i n^{\rm F}_i, \tag{S1}$$

where $c^{\dagger}_{j,\sigma}$ creates a fermion of spin $\sigma \in \uparrow,\downarrow$ at a site j, and $n^{\rm F}_j = n^{\rm F}_{j,\uparrow} + n^{\rm F}_{j,\downarrow}$ is the fermion number operator. The parameters $t^{\rm F}$ and $V^{\rm F}$ denote the nearest-neighbor fermion hopping and repulsion, respectively. $\mu^{\rm F}_j$ is the fermion chemical potential which carries a site index to allow for on-site disorder. The $U^{\rm F}$ term defines an attractive on-site interaction.

Eq. (S1) maps to an extended Bose-Hubbard model in the strongly attractive limit[S1]. We take the limit: $|U^{\rm F}| \gg t^{\rm F}, V^{\rm F}$. In this limit double occupancies are favored. We can think of each doublon as a locally formed Cooper pair. Cooper pair hopping is a second order process when viewed in terms of the original fermions. To second order in perturbation theory, Eq. (S1) reduces to[S1]:

$$H_{\rm F} \to -\bar{t}\sum_{\langle i,j\rangle}\left(\bar{b}^{\dagger}_i\bar{b}_j + {\rm H.c.}\right) + \left(\bar{t}+4V^{\rm F}\right)\sum_{\langle i,j\rangle}\bar{n}_i\bar{n}_j - \sum_j \bar{\mu}_j\bar{n}_j, \tag{S2}$$

where $\bar{b}^{\dagger}_i \equiv c_{i,\downarrow}c_{i,\uparrow}$ and $\bar{b}_i \equiv c^{\dagger}_{i,\downarrow}c^{\dagger}_{i,\uparrow}$ create and annihilate hardcore bosons, respectively. The boson number operator is then $\bar{n}_i \equiv \bar{b}^{\dagger}_i\bar{b}_i$. $\bar{t} = 2(t^{\rm F})^2/|U^{\rm F}|$ is an effective hopping of Cooper pairs. The renormalized chemical potential is: $\bar{\mu} = -2\mu^{\rm F}_j - |U^{\rm F}| + z(\bar{t}+4V^{\rm F})$.

Eq. (S2) is a disordered extended Bose-Hubbard model but there are differences between Eqs. (1) and (S2). The bosons in Eq. (S2) are hardcore but in Eq. (1) they are soft core. The two models can then be equated by taking $U \to \infty$ in Eq. (1) while also requiring a dilute limit. The latter requirement stems from the commutation relations for hardcore bosons: $[\bar{b}_i, \bar{b}^{\dagger}_j] = (1-2\bar{n}_i)\delta_{i,j}$. The commutation relation shows that Eq. (S2) maps onto Eq. (1) only for $\langle\bar{n}_i\rangle \ll 1$.

The mapping above shows that we can interpret results derived for bosons in Eq. (1) as approximations to states found in the attractive Fermi-Hubbard model. For example, the superfluid and solid order of Eq. (1) map to superconducting and charge density wave order, respectively. With this construction the supersolid state then corresponds to the coexistence of superconductivity and charge density wave orders[S1–S7]. Our analysis of Eq. (1) will therefore also offer a probe of coexisting superconductivity and charge density wave order in the presence of disorder. For example, we show that disorder actually raises the critical temperature of the supersolid for some hoppings. By appealing to the above mapping our results will therefore show that disorder increases the critical temperature of the coexisting superconductivity and charge density wave state found in Eq. (S2). This is consistent with increases in critical temperatures due to disorder found using the Bogoliubov-de Genne equations on the attractive Fermi-Hubbard model[S8].

Ising Spin Limit: The Blume-Capel Model.

The $t = 0$ limit of Eq. (1) maps into a spin-1 Ising model, the Blume-Capel model[S9,S10], at low chemical potentials. We can use this mapping to gain insight into the possible phases and scaling. This section explores the connection between the Blume-Capel model and Eq. (1).

To map Eq. (1) to a spin-1 Ising model in the dilute limit we impose a cutoff in the number of bosons per site such that n_i only takes values $0, 1$, and 2. We can then define a spin-1 Ising spin variable:

$$\sigma_i = 1 - n_i,$$

where σ_i has values $+1, 0, -1$.

The dilute $t = 0$ limit of Eq. (1) then reduces to:

$$\frac{U}{2}\sum_i \sigma_i^2 + V\sum_{\langle ij\rangle}\sigma_i\sigma_j + \sum_i(\mu_i - \frac{U}{2} - zV)\sigma_i. \tag{S3}$$

This model can be further simplified by performing a spin rotation on one of the sub lattices, i.e., $\sigma_i \to -\sigma_i$ for i on odd lattice sites. We obtain the Blume-Capel model[S9,S10]:

$$H_I \equiv \frac{U}{2}\sum_i \sigma_i^2 - V\sum_{\langle ij\rangle}\sigma_i\sigma_j, \tag{S4}$$

where we have omitted the staggered magnetic field term that derives from the chemical potential term in Eq. (1).

There are two phases in the translationally invariant Blume-Capel model at zero temperature. The ferromagnetic phase, with order parameters $\langle\sigma_i\rangle = 1$ and $\langle\sigma_i^2\rangle - \langle\sigma_i\rangle^2 = 0$, arises in the $V/U \gg 1$ limit. The non-magnetic phase, $\langle\sigma_i\rangle = 0$ and $\langle\sigma_i^2\rangle - \langle\sigma_i\rangle^2 = 1$, arises in the $V/U \ll 1$ limit. The magnetic and non-magnetic phases correspond to the solid and Mott phases in Eq. (1), respectively.

[S1] R. Micnas, J. Ranninger, and S. Robaszkiewicz, Rev. Mod. Phys. **62**, 113 (1990).
[S2] S. Robaszkiewicz, R. Micnas, and K. A. Chao, Phys. Rev. B **23**, 1447 (1981).
[S3] S. Robaszkiewicz, R. Micnas, and K. A. Chao, Phys. Rev. B **24**, 1579 (1981).
[S4] J. E. Hirsch and D. J. Scalapino, Phys. Rev. B **32**, 5639 (1985).
[S5] E. Dagotto, A. Moreo, and U. Wolff, Phys. Lett. B **186**, 395 (1987).
[S6] C. M. Varma, Phys. Rev. Lett. **61**, 2713 (1988).
[S7] R. T. Scalettar, E. Y. Loh, J. E. Gubernatis, A. Moreo, S. R. White, D. J. Scalapino, R. L. Sugar, and E. Dagotto, Phys. Rev. Lett. **62**, 1407 (1989).
[S8] K. Aryanpour, T. Paiva, W. E. Pickett, and R. T. Scalettar, Phys. Rev. B **76**, 184521 (2007).
[S9] M. Blume, Phys. Rev. **141**, 517 (1966).
[S10] H. Capel, Physica **32**, 966 (1966).
